# Predictive value of triglyceride glucose index combined with neutrophil-to-lymphocyte ratio for major adverse cardiac events after PCI for acute ST-segment elevation myocardial infarction

**DOI:** 10.1038/s41598-024-63604-9

**Published:** 2024-06-02

**Authors:** Long Wang, Yuqi Wang, Wei Wang, Zheng Wang

**Affiliations:** Department of Cardiology, The Second People’s Hospital of Hefei, Hefei Hospital Affiliated to Anhui Medical University, West Side of the Intersection of Guangde Road and Leshui Road Yaohai District, Hefei, 230000 Anhui China

**Keywords:** Acute ST-segment elevation myocardial infarction, STEMI, Triglyceride glucose index, TyG, Neutrophil-to-lymphocyte ratio, NLR, Percutaneous coronary intervention, MACE, Interventional cardiology, Acute coronary syndromes

## Abstract

Acute ST-segment elevation myocardial infarction (STEMI) is a severe cardiovascular disease that poses a significant threat to the life and health of patients. This study aimed to investigate the predictive value of triglyceride glucose index (TyG) combined with neutrophil-to-lymphocyte ratio (NLR) for in-hospital cardiac adverse event (MACE) after PCI in STEMI patients. From October 2019 to June 2023, 398 STEMI patients underwent emergency PCI in the Second People's Hospital of Hefei. Stepwise regression backward method and multivariate logistic regression analysis were used to screen the independent risk factors of MACE in STEMI patients. To construct the prediction model of in-hospital MACE after PCI in STEMI patients: Grace score model is the old model (model A); TyG combined with NLR model (model B); Grace score combined with TyG and NLR model is the new model (model C). We assessed the clinical usefulness of the predictive model by comparing Integrated Discrimination Improvement (IDI), Net Reclassification Index (NRI), Receiver Operating Characteristic Curve (ROC), and Decision Curve Analysis (DCA). Stepwise regression and multivariate logistic regression analysis showed that TyG and NLR were independent risk factors for in-hospital MACE after PCI in STEMI patients. The constructed Model C was compared to Model A. Results showed NRI 0.5973; NRI + 0.3036, NRI − 0.2937, IDI 0.3583. These results show that the newly developed model C predicts the results better than model A, indicating that the model is more accurate. The ROC analysis results showed that the AUC of Model A for predicting MACE in STEMI was 0.749. Model B predicted MACE in STEMI with an AUC of 0.685. Model C predicted MACE in STEMI with an AUC of 0.839. For DCA, Model C has a better net return between threshold probability 0.1 and 0.78, which is better than Model A and Model B. In this study, by combining TyG, NLR, and Grace score, it was shown that TyG combined with NLR could reasonably predict the occurrence of MACE after PCI in STEMI patients and the clinical utility of the prediction model.

## Introduction

Acute coronary syndrome (ACS) remains a common cause of cardiovascular death worldwide, and its incidence is increasing, including ST-segment elevation myocardial infarction (STEMI), non-ST-segment elevation myocardial infarction (NSTEMI), and unstable angina (UA)^1^. Among them, the incidence of STEMI is gradually decreasing, aggravating the global healthcare system's economic burden and seriously threatening human health and development^2^. Although the mortality rate of STEMI patients has reduced with the growth of coronary intervention (PCI), antithrombotic therapy, and secondary prevention, the incidence of myocardial infarction-related complications such as heart failure (HF) and arrhythmia has gradually increased and become an essential factor affecting the prognosis of STEMI patients^3^. Therefore, early identification, prevention, and management of complications in patients with myocardial infarction is still one of the crucial directions in the development of cardiovascular disease.

Insulin resistance (IR) has been demonstrated to be one of the risk factors for coronary atherosclerosis, and IR leads to abnormal metabolic pathways of nutrients, including blood lipids and blood glucose, by reducing the sensitivity of insulin metabolic effects; in addition, IR promotes the development of atherosclerosis by triggering pathological conditions such as inflammation, vasoconstriction and thrombosis^4,5^. Several studies have demonstrated that the serum triglyceride and glucose product (TyG) index is significantly correlated with the results of the IR gold standard-hyperinsulinemic euglycemic clamp test (HIEC), and its simplicity, economy, and ease of availability make its clinical application more effective^6^. Recent studies have shown^7,8^ that TyG index levels are associated with arterial plaque characteristics and long-term prognosis in STEMI patients, which provides evidence-based medical evidence that the TyG index may be used as a risk stratification indicator in STEMI patients. Still, there are no studies on the correlation between this index and short-term prognosis in STEMI patients.

Neutrophils and lymphocytes are classical inflammatory cells. At the onset of STEMI, the body is in a state of severe inflammation and stress response, neutrophils are recruited in large numbers, and inflammatory factors and reactive oxygen species are further released to trigger inflammatory storm and aggravate stress; inflammation and stress response activate the body's neuroendocrine system, resulting in the production and apoptosis of lymphocytes affected and decreased count levels. Therefore, at the onset of STEMI, the ratio of these two indicators, that is, neutrophil–lymphocyte ratio (NLR), can more systematically and accurately evaluate the degree of body inflammation and stress. Many high-risk factors for STEMI can place the human body in a chronic low-intensity inflammatory state for a long time, and the increase in NLR ratio when an inflammatory storm occurs may be related to the severity and prognosis of inflammation-based diseases. Past studies have reported that NLR has an excellent predictive value for the severity and prognosis of diseases such as AMI. Patients with high levels of NLR usually have more severe coronary artery disease^9^, larger infarct size^10^, worse cardiac function, high mortality, and increased risk of MACEs^11–13^.

There are few relevant studies on the predictive value of TyG combined with NLR for in-hospital MACE after PCI in STEMI patients. This study aimed to investigate the correlation between TyG combined with NLR and in-hospital MACE after PCI in STEMI patients and to clarify the predictive value of TyG combined with NLR for in-hospital MACE after PCI in STEMI patients to provide new ideas for clinical prognostic risk stratification in STEMI patients.

## Materials and methods

### Study population

We retrospectively analyzed 398 STEMI patients who underwent emergency PCI in our hospital from October 2019 to June 2023 for inclusion criteria: (1) all patients met the diagnosis and treatment criteria for acute myocardial infarction^14^; (2) all patients completed myocardial enzymes, troponin, coronary angiography, and electrocardiography, and all patients could tolerate emergency PCI; (3) aged > 18 years; and (4) patients with complete clinical data. Exclusion criteria : (1) previous myocardial infarction; (2) combined with severe metabolic system diseases; (3) combined with severe liver and kidney and other organ dysfunction; (4) combined with severe infection; (5) combined with coagulation dysfunction; (6) recent history of major surgical trauma. MACE defined the primary endpoint as cardiac death. Secondary endpoints were myocardial reinfarction, malignant arrhythmia, and acute heart failure. Diagnosis of acute heart failure: shortness of breath, orthopnea, pulmonary rales, pink foamy sputum, and other clinical manifestations; NT-proBNP: > 450 ng/L in patients under 50 years old, > 900 ng/L in patients over 50 years old, > 1800 ng/L in patients over 75 years old, > 1200 ng/L in patients with renal insufficiency (glomerular filtration rate < 60 ml/min). Malignant arrhythmias include severe sinus bradycardia (≤ 40 beats/min), high-grade or third-degree atrioventricular block, ventricular tachycardia, ventricular fibrillation, etc.,and cardiac arrest is classified as a particular type of malignant arrhythmia^15^. This study was approved by the Ethics Committee of the Second People's Hospital of Hefei(2020-KE-058).Informed consent was obtained from all participants and/or their legal guardians. Studies involving human research participants were conducted in accordance with the Declaration of Helsinki.

### Study methods

#### Data collection

Demographic characteristics and clinical data at admission were collected from STEMI patients through a hospital electronic medical record system, including age, gender, body mass index (BMI), smoking, hypertension, diabetes, systolic blood pressure (SBP), diastolic blood pressure (DBP), monocytes, red blood cells, hemoglobin, platelets, glycosylated hemoglobin, albumin, globulin, urea, creatinine, uric acid, direct bilirubin, indirect bilirubin, total cholesterol, high-density lipoprotein C (HDL-C), low-density lipoprotein C (LDL-C), left ventricular ejection fraction (LVEF).

#### Definition of TyG and NLR

Neutrophil to lymphocyte ratio (NLR) was measured after PCI. Because triglyceride glucose index (TyG) requires fasting triglycerides and glucose, TyG is a fasting measurement after PCI. Triglyceride glucose index (TyG): ln[fasting triglycerides(mg/dL) × fasting glucose(mg/dL)/2],neutrophil-to-lymphocyte ratio (NLR): neutrophils (× 10^9^/L)/lymphocytes (× 10^9^/L).

#### Study grouping

A total of 398 patients who met the above diagnostic criteria for myocardial infarction were divided into MACE group (n = 112, 28.14%) and non-MACE group (n = 286, 71.86%) according to the occurrence of MACE events; Grace score, model A, TyG combined with NLR, model B, Grace score combined with TyG and NLR model C, was calculated and divided into high and low groups according to the median.

#### Model establishment and validation

Stepwise regression backward method and multivariate logistic regression were used to analyze the independent predictors of MACE after PCI in STEMI patients. The Grace integral model of the prediction model was constructed as the old model (model A); TyG combined with NLR model (model B); Grace essential combined with TyG and NLR model as the new model (model C); and the clinical utility of the new prediction model, model C, was assessed by the net reclassification index (NRI), Integrated Discrimination Improvement (IDI), receiver operating characteristic curve (ROC), and decision curve analysis (DCA).

#### Statistical methods

Statistical analysis and plotting were performed using SPSS 26.0 and R4.2.1. Independent sample t-test was used to compare the measurement data between the two groups; enumeration data adoption rate was expressed, chi-square test was used to compare the two groups; stepwise regression backward method and multivariate logistic regression were used to analyze the independent risk factors affecting MACE after PCI in STEMI patients; NRI, IDI, the area under ROC curve (AUC) were calculated, and clinical decision curves were drawn. All statistics were performed using two-sided tests, and *P* < 0.05 was considered statistically significant.

### Ethical approval

This study was approved by the Ethics Committee of the Second People's Hospital of Hefei. Informed consent was obtained from all participants and/or their legal guardians. Studies involving human research participants were conducted in accordance with the Declaration of Helsinki.

## Results

### Comparison of baseline data and laboratory parameters between MACE group and non-MACE group

There were significant differences in age, diabetes, hypertension, SBP, DBP, monocytes, glycosylated hemoglobin, urea, creatinine, uric acid, albumin, indirect bilirubin, LVEF, TyG, and NLR levels between the MACE group and the non-MACE group (*P* < 0.05); there was no significant difference in other parameters (*P* > 0.05), as shown in Table 1.

**Table 1:** Comparison of general clinical data between MACE group and non-MACE group.

Variables	MACE(n = 112)	Non-MACE(n = 286)	*t*/*χ*^*2*^ Value	*P-*Value
Age (years)	66.14 ± 14.53	61.38 ± 13.62	3.077	0.002*
Gender (Female, n%)	80(71.4)	230(80.4)	3.778	0.052
BMI (kg/m^2^)	24.63 ± 3.48	24.35 ± 3.45	0.734	0.463
Diabetes (n%)	36(32.1)	54(18.9)	8.089	0.004*
Hypertension (n%)	78(69.6)	140(49)	13.91	< 0.001*
Smoking (n%)	58(51.8)	161(56.3)	0.661	0.416
SBP (mmHg)	113.74 ± 27.2	126.45 ± 21.92	4.415	< 0.001*
DBP (mmHg)	68.75 ± 15.9	77.38 ± 14.34	5.237	< 0.001*
Monocyte (× 10^9^/L)	0.74 ± 0.72	0.56 ± 0.27	2.531	0.013*
Red blood cell (× 10^9^/L)	4.38 ± 0.61	4.47 ± 0.6	1.306	0.192
Hemoglobin (g/L)	133.12 ± 19.79	137.19 ± 18.31	1.948	0.052
Platelets (× 10^9^/L)	207.81 ± 62.72	200.21 ± 65.92	1.048	0.295
Glycosylated hemoglobin	7.16 ± 2.19	6.35 ± 1.5	3.579	< 0.001*
Urea (mmol/L)	7.66 ± 3.58	5.52 ± 1.87	6.026	< 0.001*
Creatinine (umol/L)	99.44 ± 69.4	71.98 ± 21.26	4.113	< 0.001*
Uric acid (umol/L)	393.86 ± 120.29	352.33 ± 98.77	3.54	< 0.001*
Albumin (g/L)	38.26 ± 4.13	39.26 ± 3.68	2.279	0.023*
Globulin (g/L)	23.81 ± 4	23.16 ± 4.14	1.418	0.157
Direct bilirubin (umol/L)	5.44 ± 3.07	5.75 ± 3.03	0.915	0.361
Indirect bilirubin (umol/L)	13.14 ± 7.09	14.62 ± 7.51	1.757	0.08*
Total cholesterol (mmol/L)	4.39 ± 1.02	4.45 ± 0.94	0.629	0.53
LDL-C (mmol/L)	2.81 ± 0.87	2.84 ± 0.8	0.312	0.755
HDL-C (mmol/L)	1.12 ± 0.26	1.12 ± 0.28	0.128	0.898
LVEF	53.21 ± 11.11	58.21 ± 9.77	4.178	< 0.001*
TyG	7.54 ± 0.8	7.22 ± 0.58	3.96	< 0.001*
NLR	8.66 ± 7.11	6.17 ± 4.66	3.43	0.001*
Gensini score^b^	78.5 ± 17.74	76.5 ± 18.95	1.166	0.183
D-to-B time	63.23 ± 7.48	62.5 ± 7.32	2.586	0.247
Infarct location (n,%)			0.082	0.775
Anterior MI	55(49.1)	145(50.7)		
Others	57(50.9)	141(49.3)		
Number of diseased vessels (n,%)			0.073	0.788
1	43(38.4)	114(39.9)		
≥ 2	69(61.6)	172(60.1)		
Discharge medication (n,%)				
Aspirin	105(93.8)	264(92.3)	0.248	0.619
Clopidogrel	69(61.6)	167(58.4)	0.345	0.557
Ticagrelor	45(40.2)	127(44.4)	0.586	0.444
Statins	99(88.4)	245(85.7)	0.551	0.475
ACEI/ARB	73(65.2)	186(65)	0.001	0.978
Beta blockers	70(62.5)	193(67.5)	0.891	0.345

BMI: Body mass index; SBP: Systolic blood pressure; DBP: Diastolic blood pressure; LDH: Lactate dehydrogenase; HDL-C: High-density lipoprotein C; LDL-C: Low-density lipoprotein C; LVEF: Left ventricular ejection fraction; TyG: Triglyceride glucose index; NLR: Neutrophil-to-lymphocyte ratio.

*: P < 0.05.

### Risk factor analysis and feature selection of in-hospital MACE after PCI in STEMI patients

**Figure 1:**
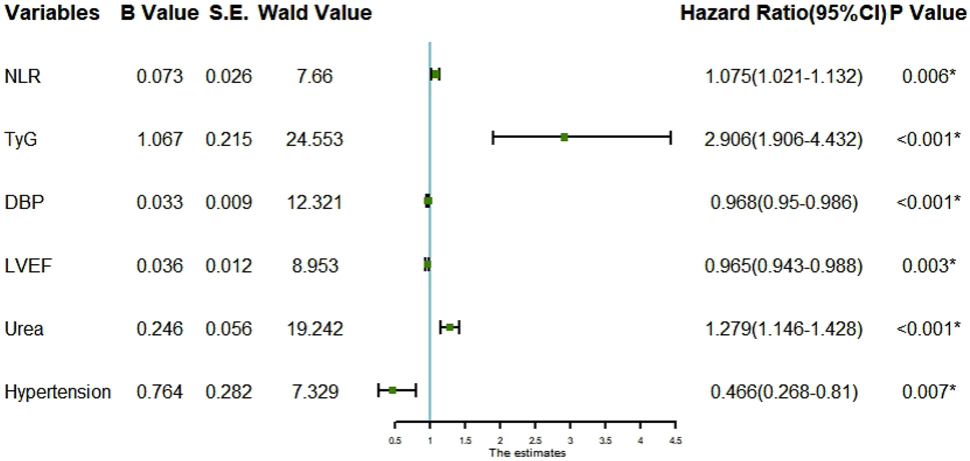
Forest plot for multivariate Logistic regression analysis;DBP:Diastolic blood pressure;;LVEF:Left ventricular ejection fraction;TyG:Triglyceride glucose index;NLR:Neutrophil-to-lymphocyte ratio.

**Figure 2:**
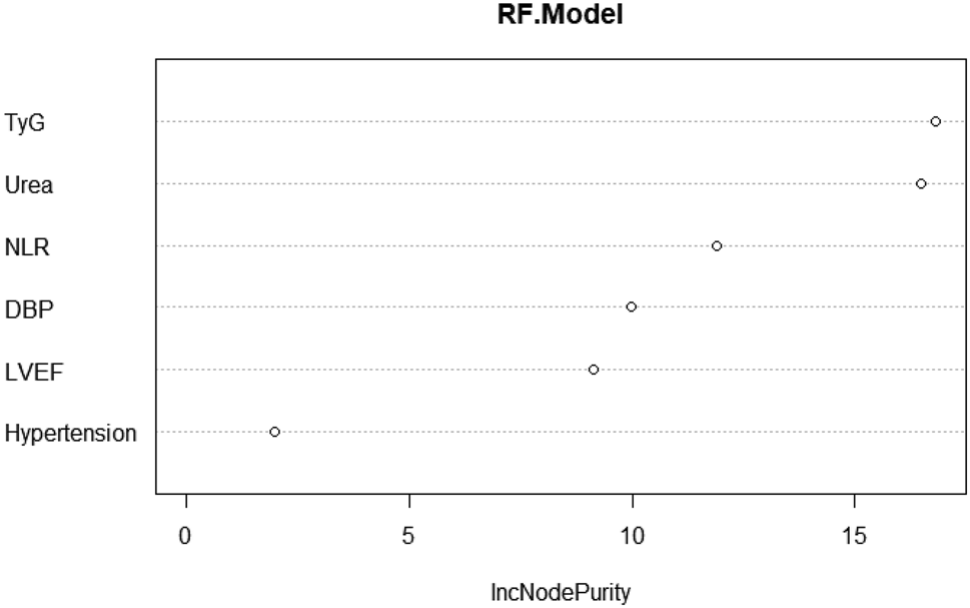
Random Forest Variable Importance Ranking;DBP:Diastolic blood pressure;;LVEF:Left ventricular ejection fraction;TyG:Triglyceride glucose index;NLR:Neutrophil-to-lymphocyte ratio.

The factors with statistically significant differences in Table 1 were used as independent variables and stepwise regression was performed with whether in-hospital MACE occurred after PCI as the dependent variable for variable screening, and finally, seven important variables, monocytes, hypertension, NLR, DBP, urea, LVEF, and TyG, were selected. Taking the significant variables screened by stepwise regression as independent variables and whether patients developed in-hospital MACE after PCI as dependent variables, multivariate logistic regression analysis revealed that hypertension, NLR, DBP, urea, LVEF, and TyG were independent risk factors for in-hospital MACE after PCI in STEMI patients (*P* < 0.05), as shown in Fig. 1. Random forest was also used to rank the importance of variables, and TyG and NLR were found to be significant independent risk factors affecting MACE after PCI in STEMI patients, as shown in Fig. 2.

### Logistic regression model to test the correlation model and the relationship between TyG, NLR, and MACE

Regression models were established, as shown in Table 2. Compared with the low group, STEMI patients in the high group had a 3.461-fold increased risk of in-hospital MACE events after PCI in model 1 (HR: 4.461, 95% CI 2.736–7.272, *P* < 0.05), 1.377-fold (HR: 2.377, 95% CI 1.51–3.741, *P* < 0.05), and 7.312-fold (HR: 8.312, 95% CI 4.802–14.388, *P* < 0.05) in model A, model B, and model C, respectively; Model 2 was statistically significant after adjusting for age, sex, BMI, diabetes, hypertension, and smoking history (HR: 5.512, 95% CI 3.024–10.049, *P* < 0.05), (HR: 2.169, 95% CI 1.347–3.493, *P* < 0.05), and (HR: 7.982, 95% CI 4.483–14.211, *P* < 0.05);Model 3 further adjusted for confounding factors such as age, sex, BMI, diabetes, hypertension, smoking history, DBP, urea, and LVEF, the risk of in-hospital MACE events after PCI increased 3.663-fold (HR: 4.663, 95% CI 2.532–8.586, *P* < 0.05), 1.169-fold (HR: 2.169, 95% CI 1.347–3.493, *P* < 0.05), and 5.785-fold (HR: 6.785, 95% CI 3.778–12.184, *P* < 0.05) in the high STEMI group, respectively.

**Table 2:** Model A, Model B and Model C predicted MACE events.

		Model 1		Model 2		Model3	
		OR(95% CI)	*P*	OR(95% CI)	*P*	OR(95% CI)	*P*
Model A	Low	Ref		Ref		Ref	
	High	4.461(2.736,7.272)	< 0.001	5.512(3.024,10.049)	< 0.001	4.663(2.532,8.586)	< 0.001
Model B	Low	Ref		Ref		Ref	
	High	2.377(1.51,3.741)	< 0.001	2.169(1.347,3.493)	0.001	2.169(1.347,3.493)	0.001
Model C	Low	Ref		Ref		Ref	
	High	8.312(4.802,14.388)	< 0.001	7.982(4.483,14.211)	< 0.001	6.785(3.778,12.184)	< 0.001

Model 1: unadjusted; Model 2: adjusted for age, sex, BMI, hypertension, diabetes, smoking history; Model 3: adjusted for age, sex, BMI, hypertension, diabetes, smoking history, DBP, urea, LVEF; Model A: Grace Score; Model B: TyG + NLR; Model C: Grace Score + TyG + NLR; They were divided into two groups according to the median: High and Low.

### Predictive value of TyG combined with NLR for in-hospital MACE after PCI in STEMI patients

**Figure 3:**
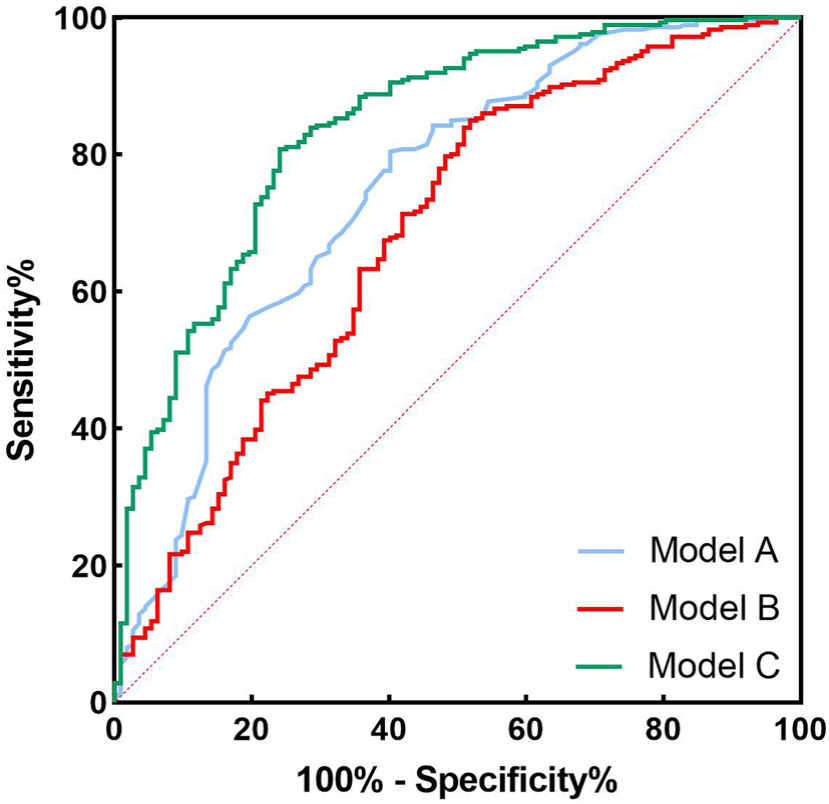
ROC curves of TyG and NLR for predicting MACE after PCI in STEMI patients;Model A: Grace Score; Model B: TyG + NLR; Model C: Grace Score + TyG + NLR.

ROC curve analysis showed that the AUC of model A for predicting MACE after PCI in STEMI patients was 0.749 (95% CI 0.693–0.805, *P* < 0.05), with a sensitivity of 0.598 and a specificity of 0.804. Model B predicted MACE in STEMI patients after PCI with an AUC of 0.685 (95% CI 0.624–0.746, *P* < 0.05), sensitivity of 0.482, and specificity of 0.85. Model C had an AUC of 0.839 (95% CI 0.794–0.883, *P* < 0.05), a sensitivity of 0.759, and a specificity of 0.808 for predicting MACE after PCI in STEMI patients, as shown in Table 3 and Fig. 3.

**Table 3:** Predictive value of TyG and NLR for MACE after PCI in STEMI patients.

	S.E	AUC (95% CI)	P	Sensitivity (%)	Specificity (%)	Youden index
Model A	0.029	0.749(0.693,0.805)	< 0.001	0.598	0.804	0.402
Model B	0.031	0.685(0.624,0.746)	< 0.001	0.482	0.85	0.332
Model C	0.023	0.839(0.794,0.883)	< 0.001	0.759	0.808	0.567

Model A: Grace Score; Model B: TyG + NLR; Model C: Grace Score + TyG + NLR.

### Comparison of clinical predictive model and GRACE Score

The accuracy of the predictive model can be assessed by the net reclassification index (NRI). If the NRI exceeds 0, it means that the new model is better than the old model, while negative values indicate the opposite. The calculation of Integrated Discriminant Improvement (IDI) is based on the predicted probability of the disease model for each individual. IDI represents the change in the gap between the predictions of the two models. Overall, it is suggested that the larger the IDI, the stronger the predictive power of the new model. If IDI > 0, it is an improvement; if IDI < 0, it is a negative improvement; if IDI = 0, there is no improvement in the new model. NRI represents the net reclassification index for all available data; NRI + reflects the net reclassification index for data where MACE occurred, and NRI – reflects the net reclassification index for data where MACE did not happen. The results showed that the new model, model C, was compared with the old model, model A: NRI, NRI + , NRI − , and IDI values were 0.5973, 0.3036, 0.2937, and 0.3583, respectively. The 95% confidence interval did not include 0, *P* < 0.001, as shown in Table 4. showed that new prediction models for specific populations are superior to GRACE scores.

**Table 4:** Comparison of Clinical Predictive Models and GRACE Score.

	Estimate	S.E	95% CI	*P*
NRI	0.5973	0.0845	0.2911–0.6312	< 0.001
NRI +	0.3036	0.0672	0.0716–0.3177	< 0.001
NRI-	0.2937	0.0662	0.1513–0.3771	< 0.001
IDI	0.3583	0.0446	0.2198–0.3161	< 0.001

NRI: Net Reclassification Improvement; IDI: Integrated Discrimination Improvement; NRI + : Reflects the net reclassification index for data where MACE occurred; NRI: Reflects the net reclassification index for data where MACE did not occur.

### Clinical Decision Analysis

**Figure 4:**
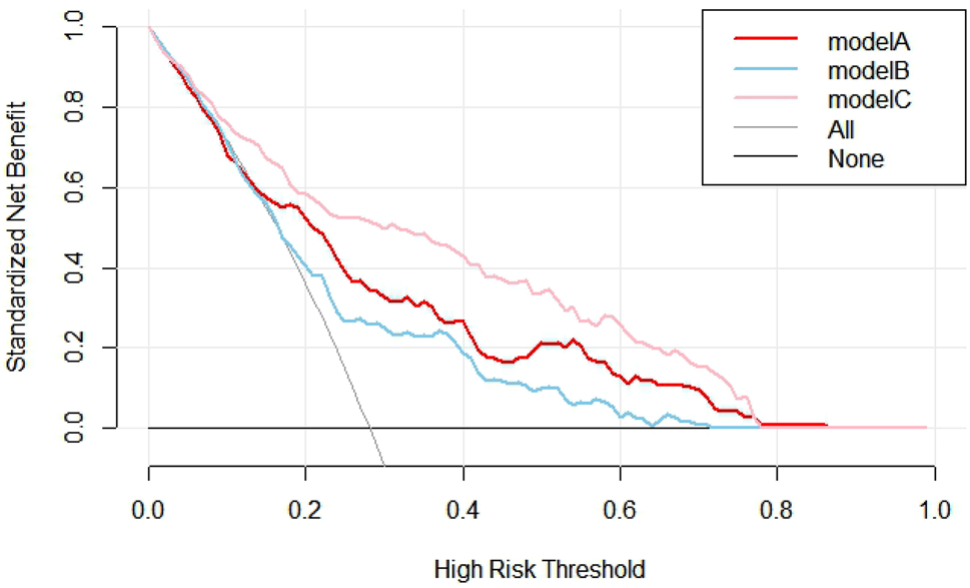
Decision curve analysis.

Figure 4 shows the decision curves of Model A, Model B, and Model C for predicting MACE after PCI in patients with STEMI. All models were effective between threshold probabilities of 0.1–0.78, and the net benefit of model C was better than that of model A and model B in the range of threshold probabilities of 0.1–0.78. These results suggest that when the threshold probability is 0.1–0.78, the new model, model C, can bring net clinical benefit to patients.

## Discussion

The prevalence and mortality of cardiovascular disease (CVD) continue to rise, and is one of the diseases threatening the health of Chinese people for a long time, of which the number of hospitalizations for acute ST-segment elevation myocardial infarction (STEMI) accounts for 86.8% of acute myocardial infarction (AMI), characterized by rapid onset, rapid progression, many complications, high mortality, and poor prognosis, and patients suffering from STEMI are more likely to develop major adverse cardiovascular events such as heart failure,high thrombus burden causing reinfarction and coronary microcirculation disturbance^16,17^, cardiogenic shock, stroke, and cardiac arrest than patients with other myocardial infarction types^18,19^. Although the establishment of chest pain centers and the workflow have continuously improved in various regions, the main means of treating the disease, percutaneous coronary intervention (PCI) treatment techniques, continue to mature. Although most patients can restore myocardial blood perfusion earlier and more timely than before, the in-hospital mortality rate of patients remains high. A significant number of patients develop MACE during hospitalization^19^.

During insulin resistance, the physiological utility of insulin is reduced, which leads to metabolic disorders, including blood lipids and blood glucose, triggers metabolic syndrome, and induces the progression of atherosclerosis through a variety of mechanisms, which has a strong correlation with cardiovascular risk^20–22^ and has become one of the hotspots of concern in the cardiovascular field. Kelly et al.^23^ demonstrated that serum TG levels were negatively correlated with insulin sensitivity, suggesting that the homeostasis between lipid metabolism and insulin efficacy is disrupted with increasing TG levels. Sanchez-Garcia A et al.^24^ showed that the TyG index had a sensitivity of 96% in the diagnosis of IR in a meta-analysis of 69,922 patients in 15 studies. With the continuous deepening of TyG index studies, TyG index has been confirmed to have a strong association with the gold standard test for the diagnosis of IR. Its predictive power is higher than the IR homeostasis assessment model^25,26^. TyG index is a simple index to evaluate IR. Several studies have confirmed that the TyG index is significantly correlated with the occurrence and prognosis of atherosclerosis^27^, and its predictive value for the prognosis of patients with coronary heart disease and type 2 diabetes is higher than that of glycosylated hemoglobin^28^.

Several studies have confirmed that the TyG index is an independent predictor of the occurrence and prognosis of cardiovascular disease^26,28,29^, but there is no conclusion on the cut-off value.Triglyceride-glucose indices have also previously been associated with slow coronary flow, which may also contribute to MACE events in patients during hospitalization^30^.Wang et al.^31^ showed in a 3-year observational study of 2531 ACS patients with diabetes that the TyG index, as independent of known traditional cardiovascular risk factors, had an optimal cut-off value for predicting MACE of 9.323, a sensitivity of 46.0%, and a specificity of 63.6% (95% CI 1.201–1.746, *P* < 0.05), and its predictive efficacy was not affected by treatment modality. Jin et al.^32^ conducted a clinical follow-up observation study of 1282 patients with stable coronary heart disease. The results showed that the TyG index level was directly proportional to the risk of cardiovascular events. The danger of cardiovascular events increased by 21.2% for each standard deviation increase in the TyG index (HR = 1.212, 95% CI 1.075–1.366, *P* < 0.05).

Studies have shown that reactive immune responses mediated by neutrophils and adaptive immune responses mediated by lymphocytes run through atherosclerotic plaque rupture. After STEMI, neutrophils infiltrate into the myocardial infarction area, produce proteolytic enzymes, and oxidize vascular endothelial cells, resulting in a hypercoagulable state of the blood; at the same time, the stress response after myocardial infarction increases cortisol hormone secretion, resulting in a decrease in the number of lymphocytes, and NLR can reflect the balance between these two inflammatory cells and more accurately reflect the inflammatory state of the body than a single cell type, with a better predictive value^33^.

High NLR has also been associated with adverse events in multiple long-term follow-up clinical studies^34^. Klein^33^ et al. included 1892 STEMI patients from various centers and divided NLR at admission into quartiles. The fourth quartile of NLR was found to be significantly associated with shock within 30 days (OR = 3.64, 95% CI 2.02–6.54). Park et al.^35^ found that patients who died within five years had a higher initial NLR (6.398 vs. 4.231, *P* = 0.004) in 326 PCI-treated STEMI patients. By regression analysis, patients with higher NLR were more likely to have MACE (OR = 1.085, 95% CI 1.002–1.174). A 6-year study of 6560 STEMI patients found a significant increase in cardiovascular mortality in the high NLR (> 3.9) group (*P* < 0.0001) by dividing the NLR into three equal points, and multivariate regression analysis found an independent predictor of annual mortality in the high NLR group (OR = 2.85, 95% CI 1.54–5.26)^36^. Another study of elderly patients with coronary heart disease divided the NLR into four quartiles in 345 patients who were robust, reasonable, and weak and found a positive correlation between physical condition and NLR grade (r = 0.169), and multivariate logistic regression analysis also found that patients in the fourth quartile of the NLR were more likely to have adverse MACE events (OR = 2.894, *P* = 0.011)^37^.

In this study, we found that TyG (OR: 2.906, 95% CI 1.906–4.432) and NLR (OR: 1.075, 95% CI 1.021–1.132) were independent risk factors for in-hospital MACE after PCI in STEMI patients by stepwise regression screening and multivariate logistic regression analysis (*P* < 0.05).

We further divided the study into three models, namely the Grace score model (Model A), TyG combined with the NLR model (Model B), and Grace score combined with the TyG and NLR model (Model C).Model C was found to have a higher risk of MACE than Model A and Model B by adjusting for confounding factors.It is further indicated that the new model composed of TyG and NLR, model C, can further improve the risk prediction ability of the model for in-hospital MACE after PCI in STEMI patients by adding TyG to the old model with Grace score.

We further evaluate the new model's accuracy, Model C, by the net reclassification index (NRI) and integrated discriminant improvement (IDI).The results showed that the new model, model C, was compared with the old model, model A: NRI, NRI + , NRI − , and IDI values were 0.5973, 0.3036, 0.2937, and 0.3583, respectively. With 95% confidence intervals excluding 0 and *P* values < 0.001, our study further suggests that Model C is superior to the GRACE score, Model A, for the occurrence of in-hospital MACE after PCI in STEMI patients.

The area under the curve (AUC) for predicting MACE after PCI in STEMI patients using the traditional model consisting of the Grace score, Model A, was 0.749. In the traditional diagnostic model composed of Grace integral, TyG and NLR are introduced to form a new diagnostic model, namely Model C. In the new diagnostic model, ROC results showed that the AUC of the three combinations for predicting MACE after PCI in STEMI patients was 0.839. The results showed that model C was superior to model A in predicting MACE after PCI in STEMI patients and had particular significance in guiding clinical work.

In parallel, we performed DCA to assess the performance of the diagnostic model. Model C showed a better net gain than Model A, with threshold probabilities of 0.1–0.78. It further illustrates that Model C is superior to Model A in predicting the occurrence of MACE after PCI in STEMI patients.

However, this study has some limitations: First, it is a single-center study with a small sample size, and it is necessary to expand the sample size and combine multi-center for further research and analysis. Second, the TyG index measured in this study was calculated within 24 h after admission. Its changes were not dynamically monitored after the application of lipid-lowering drugs. If the trial conditions allowed in the future, it could be observed at multiple points to reveal its dynamic evolution level affecting the occurrence of MACE in STEMI patients in the hospital.Third, this study did not collect the medication of patients before admission, and the medication of patients before admission should be further collected in detail at a later stage to further conduct a relevant study on the prognosis of patients.

## Conclusion

In conclusion, the results of this study suggest that TyG and NLR are associated with the occurrence of in-hospital MACE after PCI in STEMI patients and can be used as predictors of in-hospital MACE after PCI in STEMI patients. Combined with the traditional predictive model Grace score, it has higher diagnostic value, stronger sensitivity, and specificity and can be used for risk stratification and guiding individualized treatment of in-hospital MACE after PCI in STEMI patients, thereby improving the prognosis of STEMI patients.

## Data Availability

The data that support the findings of this study are not openly available due to reasons of sensitivity and are available from the corresponding author upon reasonable request.

## Abbreviations

STEMI: ST-segment elevation myocardial infarction
NSTEMI: Non-ST-segment elevation myocardial infarction
UA: Unstable angina
HF: Heart failure
IR: Insulin resistance
MACE: Major adverse cardiac events
IDI: Integrated Discrimination Improvement
NRI: Net Reclassification Index
ROC: Receiver Operating Characteristic Curve
DCA: Decision Curve Analysis
PCI: Percutaneous coronary intervention
ACS: Acute coronary syndrome
BMI: Body mass index
SBP: Systolic blood pressure
DBP: Diastolic blood pressure
LDH: Lactate dehydrogenase
HDL-C: High-density lipoprotein C
LDL-C: Low-density lipoprotein C
LVEF: Left ventricular ejection fraction
TyG: Triglyceride glucose index
NLR: Neutrophil-to-lymphocyte ratio
